# AKT Signaling as a Novel Factor Associated with *In Vitro* Resistance of Human AML to Gemtuzumab Ozogamicin

**DOI:** 10.1371/journal.pone.0053518

**Published:** 2013-01-08

**Authors:** David B. Rosen, Kimberly H. Harrington, James A. Cordeiro, Ling Y. Leung, Santosh Putta, Norman Lacayo, George S. Laszlo, Chelsea J. Gudgeon, Donna E. Hogge, Rachael E. Hawtin, Alessandra Cesano, Roland B. Walter

**Affiliations:** 1 Nodality Inc., South San Francisco, California, United States of America; 2 Clinical Research Division, Fred Hutchinson Cancer Research Center, Seattle, Washington, United States of America; 3 Division of Pediatric Hematology/Oncology, Stanford University School of Medicine, Palo Alto, California, United States of America; 4 Terry Fox Laboratory, British Columbia Cancer Agency, Vancouver, British Columbia, Canada; 5 Division of Hematology/Department of Medicine, University of Washington, Seattle, Washington, United States of America; 6 Department of Epidemiology, University of Washington, Seattle, Washington, United States of America; University of North Carolina at Chapel Hill, United States of America

## Abstract

Gemtuzumab ozogamicin (GO), an immunoconjugate between an anti-CD33 antibody and a calicheamicin-γ_1_ derivative, induces remissions and improves survival in a subset of patients with acute myeloid leukemia (AML). As the mechanisms underlying GO and calicheamicin-γ_1_ resistance are incompletely understood, we herein used flow cytometry-based single cell network profiling (SCNP) assays to study cellular responses of primary human AML cells to GO. Our data indicate that the extent of DNA damage is quantitatively impacted by CD33 expression and drug efflux activity. However, although DNA damage is required for GO-induced cytotoxicity, it is not sufficient for effective cell kill, suggesting that downstream anti-apoptotic pathways may function as relevant resistance mechanisms. Supporting this notion, we found activated PI3K/AKT signaling to be associated with GO resistance *in vitro* in primary AML cells. Consistently, the investigational AKT inhibitor MK-2206 significantly sensitized various human AML cells to GO or free calicheamicin-γ_1_ with particularly pronounced effects in otherwise GO or free calicheamicin-γ_1_ -resistant cells. Likewise, MK-2206 also sensitized primary AML cells to calicheamicin-γ_1_. Together, our findings illustrate the capacity of SCNP assays to discover chemotherapy-related biological pathways and signaling networks relevant to GO-induced genotoxic stress. The identification of AKT signaling as being associated with GO resistance *in vitro* may provide a novel approach to improve the *in vivo* efficacy of GO/calicheamicin-γ_1_ and, by extrapolation, other DNA damage-based therapeutics.

## Introduction

Most patients with acute myeloid leukemia (AML) are currently expected to die from their disease or treatment-related toxicities [1]–[4]. The need to develop effective yet well-tolerated new therapies is therefore unquestioned. Monoclonal antibodies have raised high expectations to accomplish this goal, with gemtuzumab ozogamicin (GO), an immunoconjugate between a humanized anti-CD33 antibody and a toxic calicheamicin-γ_1_ derivative [5], being the clinically most widely exploited thus far.

Emerging data indicate that GO benefits a subset of AML patients. Initial phase 2 studies found an overall response rate of ∼30% with GO monotherapy in relapsed adult AML and led to accelerated marketing approval in the U.S. in 2000 [5]. More recently, several large trials have suggested a clinical benefit when GO is added to standard chemotherapy for adults with newly diagnosed AML, in particular for those with favorable-risk and possibly intermediate-risk disease [6]–[9]. Nevertheless, GO is ineffective in many patients with AML. In fact, the lack of pre-specified overall improvement in outcome, together with increased early mortality in the GO-containing arm, resulted in premature termination of the confirmatory randomized phase 3 trial conducted by the Southwest Oncology Group (SWOG; S0106) and, subsequently, withdrawal of the new drug application in the U.S. in 2010 [10]. Overall, this experience highlights the importance of understanding the factors associated with GO resistance to optimize the clinical use of this antibody-drug conjugate.

**Figure 1:**
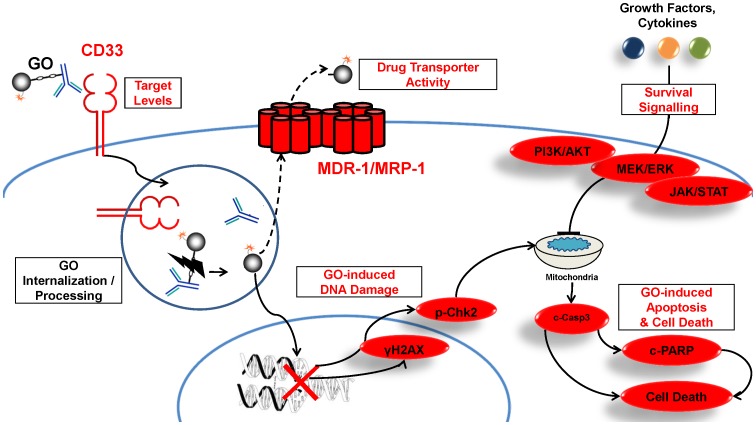
Functional characterization of GO-induced cytotoxicity in CD33^+^ cells. Scheme depicts the presumed mechanism of action of GO in CD33^+^ AML cells [5], [11]. Experimentally measured components in our studies are shown in red: CD33 expression levels; drug efflux pump activity; γH2AX (extent of DNA damage); survival signaling pathways (PI3K/AKT, MEK/ERK, and JAK/STAT pathway); and cleaved PARP and cell membrane integrity (apoptosis/cell death).

With GO, the antibody primarily facilitates cellular uptake of the calicheamicin-γ_1_ derivative, which is then released intracellularly and causes single- and double-stranded DNA damage (Figure 1) [5]. Conceptually, the amount of intracellular, active calicheamicin is affected by cellular uptake, toxin release and activation, as well as drug inactivation/metabolism or extrusion. Indeed, correlative and *in vitro* studies have shown that drug efflux mediated by P-glycoprotein (Pgp, MDR1) and, to a lesser degree, multidrug resistance protein (MRP1), mediate resistance to GO [5], [11]. Similarly consistently, experimental studies revealed a striking, quantitative relationship between CD33 expression/uptake and GO efficacy in engineered AML cell lines [12], [13].

Conversely, the impact of DNA repair and downstream signaling pathways on GO-mediated cytotoxicity has not been examined in detail. To this end, single cell network profiling (SCNP) assays using multiparametric flow cytometry have emerged as a versatile tool to simultaneously study several specific biological pathways and signaling networks at the single cell level within the context of complex tissues (e.g., blood or bone marrow) without the need for isolation or purification of the cell populations of interest [14], [15]. By characterizing cellular signaling responses following exposure to extrinsic modulators, signaling network integrity can be tested and dysfunctional properties that may not manifest in resting cells detected. We have recently demonstrated that dynamic single cell network profiles can serve as independent predictors of response to standard induction chemotherapy in newly diagnosed adult AML [16]. Herein, we demonstrate the usefulness of SCNP assays as a discovery tool to identify signaling pathways associated with responsiveness or resistance of primary AML specimens to GO *in vitro* and, consequently, novel pharmacological approaches that can overcome GO resistance.

## Materials and Methods

### Ethics Statement

In accordance with the Declaration of Helsinki, all patients (or guardians on behalf of the pediatric patients) provided written informed consent for the collection and use of their biospecimens for research purposes under studies approved by the Stanford University Research Compliance Office (Stanford, CA, USA), the British Columbia Cancer Agency Research Ethics Board (Vancouver, BC, Canada), and the Fred Hutchinson Cancer Research Center Institutional Review Board (FHCRC; Seattle, WA, USA). Clinical data were de-identified in compliance with Health Insurance Portability and Accountability Act regulations.

### Primary Human Specimens

Pretreatment (“diagnostic”) peripheral blood or bone marrow specimens were obtained from patients with newly diagnosed AML treated at the British Columbia Cancer Agency, Lucile Packard Children’s Hospital (Stanford University), or FHCRC. Normal peripheral blood mononuclear cells (PBMCs) were commercially obtained from a healthy blood donor (Blood Center, Stanford University).

### Human AML Cell Lines

U937 [17] and KG-1 [18] cells were maintained in RPMI 1640/10% fetal bovine serum (FBS; HyClone, Waltham, MA, USA). GDM-1 cells [19] were maintained in the same media supplemented with GM-CSF (2 ng/mL; R&D, Minneapolis, MN, USA). HL-60 [20], NB4 [21], and TF-1 [22] cells were maintained as previously described [23].

### Flow Cytometric SCNP Assays in Primary AML Cells

SCNP assays were performed as described previously [24]. Briefly, aliquots of cryopreserved cells were thawed at 37°C, washed, resuspended in RPMI 1640/60% FBS, and mononuclear cells isolated via ficoll density gradient. After sequential washing with RPMI 1640/60% FBS and RPMI 1640/1% FBS, cells were stained with amine aqua viability dye (see Table S1 for a complete list of reagents used and manufacturers) to distinguish viable from non-viable cells. Cells were then re-suspended in RPMI 1640/10% FBS and aliquoted at 100,000 cells/condition. Following a resting period of 1–2 hours at 37°C, cells were subjected to apoptosis or signaling pathway assays as described in detail below. Subsequently, cells were fixed with 1.6% paraformaldehyde for 10 minutes at 37°C, pelleted, permeabilized with 100% ice-cold methanol, and stored at −80°C. Cells were washed with FACS buffer (PBS, 0.5% BSA, 0.05% NaN_3_), pelleted, and stained with cocktails of fluorochrome-conjugated antibodies. These cocktails included antibodies against cell surface markers for cell population gating (e.g. CD11b, CD33, CD34, and CD45) and up to 3 antibodies against intracellular signaling molecules for an 8-color flow cytometry assay. In these analyses, CD11b, CD34, and CD45 were consistently used in the SCNP assays while some specific conditions additionally included measurements of CD33 expression. Side-scatter properties and CD45 and/or CD33 expression were used to define the leukemic cell population. Antibodies against CD11b and CD34 were included to allow for further characterization of leukemic cell subsets but were not utilized for the analyses presented in this manuscript. Isotype controls or phosphopeptide blocking experiments were performed to characterize phospho-antibodies. Data was acquired on an LSR II and/or CANTO II flow cytometers using the FACS DIVA software (BD Biosciences). All flow cytometry data were analyzed with FlowJo (TreeStar Software, Ashland, OR, USA) or WinList (Verity House Software, Topsham, ME, USA). Dead cells and debris were excluded by forward and side scatter properties combined with amine aqua viability dye exclusion. Leukemic cells were identified based side-scatter properties and expression of CD45 and/or CD33 as previously described [24], [25], and analyzed for CD33 expression levels, drug-induced DNA damage and cytotoxicity, and modulator-induced signaling as follows:

#### Quantification of CD33 expression

In pediatric specimens, CD33 was quantified using in-house biotin-conjugated CD33 followed by streptavidin-tagged Qdot605. In all adult specimens except AML-11, in which CD33-biotin-Qdot605 was not used because of limiting cell numbers, CD33 was quantified using PE-conjugated CD33, and CD33-biotin-Qdot605. To allow direct comparisons of CD33 expression levels between all specimens used in our studies, a linear regression model was construed between PE-conjugated and Qdot605-biotin-conjugated CD33 mean fluorescence intensities (MFIs), using AML samples and peripheral blood and bone marrow mononuclear cells from normal donors from our internal database with available data on both fluorophores, allowing calculation of equivalent Qdot605 MFI in specimen AML-11 using the equation: Qdot605 CD33 MFI = 0.6927×(CD33 PE MFI) +176.05 (r = 0.94; see Figure S1).

#### Quantification of drug-induced DNA damage and cytotoxicity

Following incubation with GO (30 ng/mL, expressed as free calicheamicin-γ_1_ weight equivalents; Pfizer, New York, NY) with or without the drug efflux inhibitor, PSC833 (2 µM), for 6–48 hours or incubation with staurosporine (2.33 µg/mL) for 6 hours, cells were restained with amine aqua viability dye prior to fixation and SCNP processing. Phosphorylation of histone H2AX at S139 (γH2AX) was used to quantify the extent of DNA damage in leukemic cells [26], [27], whereas antibodies detecting cleaved PARP (cPARP[Asp214]) or cleaved caspase-3 (active caspase-3) and amine aqua viability dye were used to detect apoptotic and dead cells, respectively. The percentage of leukemic “induced apoptotic/dead cells”, gated on leukemic/myeloid cells based on SSC and CD45/CD33 properties, was calculated as: [Live_Untreated_ – Live_Drug treated_]_/_[Live_Untreated_], with “live” cells defined as cPARP^−/^aqua dye^−^. This metric ranges from values of 0 to 100% for each sample and normalizes for differences in sample quality or spontaneous apoptosis. The extent of GO-induced DNA damage was calculated as: % γH2AX^+^_Drug treated_ – % γH2AX^+^_Untreated_.

#### Assessment of growth factor/cytokine-induced signaling in primary AML cells

Cells were incubated for 5–15 min at 37°C with the following modulators: FLT3 ligand (FLT3L; 50 ng/mL), IL-27 (50 ng/mL), PMA (400 nM), and SCF (20 ng/mL). Induced signaling responses were then measured using intracellular signaling molecules (p-AKT [S473], p-S6 [S235/236], p-ERK 1/2 [T202/204], p-STAT1 [Y701], p-STAT3 [Y705], and p-STAT5 [Y694]) and quantified with the Log_2_Fold metric: Log_2_ [MFI_Modulated/_MFI_Unmodulated_]. Additional details for modulation conditions and antibody cocktails used in SCNP signaling assays can be found in Table S2.

### SCNP Assessment of Kinetics of DNA Damage in Human AML Cell Lines and Normal PBMCs

For kinetic assessment of GO induced DNA damage, human AML cell lines and normal PBMCs were incubated for 1–8 h with GO (30 ng/mL) with or without the drug efflux inhibitor, cyclosporin A (2.5 µg/mL), and processed as described above.

### Western Blot Analysis of MK-2206-mediated Inhibition of AKT Signaling

Human AML cell lines were treated with MK-2206 (1 µM; partially provided by Merck & Co, Inc.; Whitehouse Station, NJ, and partially obtained from Selleck Chemicals, Houston, TX) or vehicle (DMSO) for 6 hours at 37°C in 5% CO_2_ and air. The cells were then lysed in RIPA lysis buffer with Complete EDTA-free protease inhibitor cocktail (Roche, Indianapolis, IN, USA) on ice for 60 minutes and pre-cleared by centrifugation. 4× SDS sample buffer was added to RIPA soluble proteins to a final concentration of 1× SDS sample buffer. Samples were boiled, resolved by SDS-12%-PAGE, transblotted to nitrocellulose, and blots blocked with 5% non-fat dry milk in Tris-buffered saline. Blots were incubated with primary antibodies detecting p-AKT (S473) or total AKT (both CST) overnight at 4°C. After washing, incubation with HRP-conjugated secondary antibody for 1 hour at room temperature, and re-washing, immunoreactive signals were visualized with Amersham ECL Plus Western Blotting Detection System (GE Healthcare Biosciences, Pittsburgh, PA, USA) and exposed to autoradiography film (Genesee Scientific, San Diego, CA, USA).

### Assessment of MK-2206-modulated GO and Free Calicheamicin-γ_1_-induced Cytotoxicity in AML Cell Lines and Primary AML Cells

In experiments with human AML cell lines, cells were incubated with increasing concentrations of GO or free calicheamicin-γ_1_ (kindly provided by Wyeth-Ayerst [now Pfizer]) in the presence or absence of various doses of MK-2206. To partially reverse efflux-mediated drug resistance, parallel GO-treated cultures of KG-1 cells were treated with a low, subsaturating concentration (25 µM) of the drug efflux inhibitor, PK11195 (Sigma Aldrich, St. Louis, MO, USA) [12], [28], [29]. In experiments with primary AML specimens, thawed aliquots of density gradient-separated mononuclear cells were incubated with increasing concentrations of calicheamicin-γ_1_ in the presence or absence of MK-2206. After 3 days at 37°C in 5% CO_2_ and air, cell numbers and drug-induced cytotoxicity was determined by flow cytometry using 4',6-diamidino-2-phenylindole (DAPI, Sigma Aldrich) to identify dead cells.

### Statistical Considerations

Data on drug-induced cytotoxicity and DNA damage as well as SCNP are expressed as mean ± SEM. Data on GO-induced cytotoxicity and DNA damage were correlated with CD33 expression levels using Pearson’s linear correlation and expressed as correlation coefficient [95% confidence interval]. Two-way ANOVA for repeated measures was used to analyze cytotoxicity data in AML cell lines, which are shown as percent change in dead cells and cell numbers relative to parallel cultures treated without GO or free calicheamicin-γ_1_ but with corresponding amounts of MK-2206. All statistical analyses were performed with Prism or InStat (Graphpad, San Diego, CA); *P*<0.05 was considered statistically significant.

## Results

### Basic Characterization of in vitro Sensitivity of Primary AML Specimens to GO

**Figure 2:**
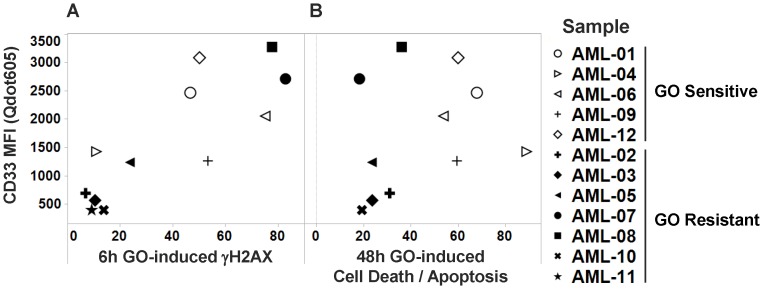
Relationship between CD33 level and GO-induced DNA damage and cytotoxicity. (**A**) Correlation between CD33 expression and GO-induced DNA damage, as quantified by measurement of γH2AX levels 6 hours after drug exposure. (**B**) Correlation between CD33 expression and GO-induced cytotoxicity after 48 hours of drug exposure. Samples resistant to GO (as defined by <15% GO-induced apoptosis/cell death 24 hours after drug exposure) are shown in bold. Note: the CD33-QDot605 MFI for AML-11 was converted from CD33 PE MFI, as described in Materials and Methods.

The postulated mechanism of action of GO implies a pivotal role of drug-induced DNA damage and intact downstream pro- and anti-apoptotic pathway activity [5]. As depicted in Figure 1, our SCNP assay focused on functional, quantitative measurements of DNA damage pathways as well as PI3K/AKT, MEK/ERK, and JAK/STAT signaling pathways in leukemic cells in response to *in vitro* stimulation of diagnostic specimens from 6 pediatric and 6 adult patients containing >78% blast cells in all cases (Table 1; AML-01– AML-12). First, the *in vitro* GO sensitivity of AML cells in these specimens was characterized. At the clinically relevant concentration of 30 ng/mL [30], GO treatment resulted in an induced apoptotic/dead cell fraction averaging 19.3±5.2% and 43.8±7.1% after continuous exposure for 24 and 48 hours, respectively (Table 2). Using an arbitrary cut-off for the induced apoptotic/dead cell fraction of 15% at 24 hours, 5 specimens were classified as “GO sensitive”, whereas 7 were classified as “GO resistant”. The lack of apoptotic response in these GO resistant samples was likely directly due to insufficient intracellular GO concentrations rather than the consequence of dysfunctional apoptotic pathways, as parallel experiments with staurosporine, a classic activator of the mitochondrial apoptosis pathway [31], demonstrated robust apoptosis not only in GO-sensitive but also in most (5/6 tested) GO resistant samples (Figure S2), establishing that the apoptotic machinery was generally intact in the tested AML specimens. Of note, among all patient specimens, there was no correlation between CD33 expression levels and GO-induced cytotoxicity at 24 hours (r = 0.181 [95% confidence interval: −0.439–0.684], *P* = 0.574) or 48 hours (r = 0.266 [−0.397–0.747], *P* = 0.429). Importantly, GO failed to induce significant cytotoxicity after 24 or 48 hours in 2 cases (AML-07 and AML-08) with bright CD33 expression, indicating the presence of relevant resistance mechanisms that are unrelated to target antigen expression (Figure 2).

**Table 1:** Primary AML Patient and Specimen Characteristics.

Specimen	Category	Age (years)	Gender	Cytogenetics	FLT3 Mutational Status	Tissue Source	% Blasts^1^
AML-01	Pediatric	12	Male	der(3)t(1;3)	WT	BM	91
AML-02	Pediatric	17.5	Male	NK	WT	BM	83
AML-03	Pediatric	15.8	Male	NK	ITD	BM	86
AML-04	Pediatric	15.7	Male	NK	ITD	BM	86
AML-05	Pediatric	2.6	Male	NK	WT	BM	88
AML-06	Pediatric	14.6	Female	NK	ITD	BM	92
AML-07	Adult	67	Male	NK	Unknown	PB	91
AML-08	Adult	67	Male	NK	Unknown	BM	95
AML-09	Adult	44	Male	NK	WT	PB	94
AML-10	Adult	47	Female	t(9;11)(p22;q23)	Unknown	PB	85
AML-11	Adult	33	Female	NK	WT	PB	79
AML-12	Adult	63	Female	NK	ITD	PB	93
AML-13	Adult	78	Male	NK	WT	BM	80
AML-14	Adult	83	Female	Complex	Unknown	PB	38
AML-15	Adult	83	Unknown	Unknown	Unknown	PB	Unknown

**Abbreviations:** BM, bone marrow; ITD, internal tandem duplication; NK, normal karyotype; PB, peripheral blood; WT, wild-type.

1 % Blasts among viable mononuclear cells.

**Table 2:** GO-induced Apoptosis/Cell Death and γH2AX in Primary AML Specimens.

Specimen	CD33 (MFI)*	GO-induced γH2AX (%)		GO-inducedApoptosis/Cell Death (%)		CytotoxicResponse
	6 hours	6 hours	24 hours	24 hours	48 hours	
AML-10	398	13.7	23.9	9.9	19.3	Resistant
AML-11	409	9.1	6.9	9.9	NA	Resistant
AML-03	569	10.4	66.8	8.9	23.7	Resistant
AML-02	695	6.8	4.4	7.6	31.0	Resistant
AML-05	1,240	23.6	45.0	11.3	23.5	Resistant
AML-07	2,713	82.8	80.0	0	18.3	Resistant
AML-08	3,274	77.7	50.7	0	36.1	Resistant
AML-09	1,265	53.3	33.7	27.0	59.4	Sensitive
AML-04	1,429	10.9	23.6	35.5	88.9	Sensitive
AML-06	2,057	75.1	74.0	38.2	53.7	Sensitive
AML-01	2,468	46.6	35.8	59.6	67.8	Sensitive
AML-12	3,086	50.1	33.4	23.7	59.9	Sensitive

**Abbreviations:** MFI, mean fluorescence intensity; NA, not available (insufficient sample material).

* CD33 MFI are shown for the Qdot605 fluorophore scale. For AML-11, this value was converted from CD33-PE expression due to insufficient sample for CD33-Qdot605 assessment (see “Materials and Methods”).

### Extent of DNA Damage Correlates with CD33 Expression Levels but not GO-induced Cytotoxicity

**Figure 3:**
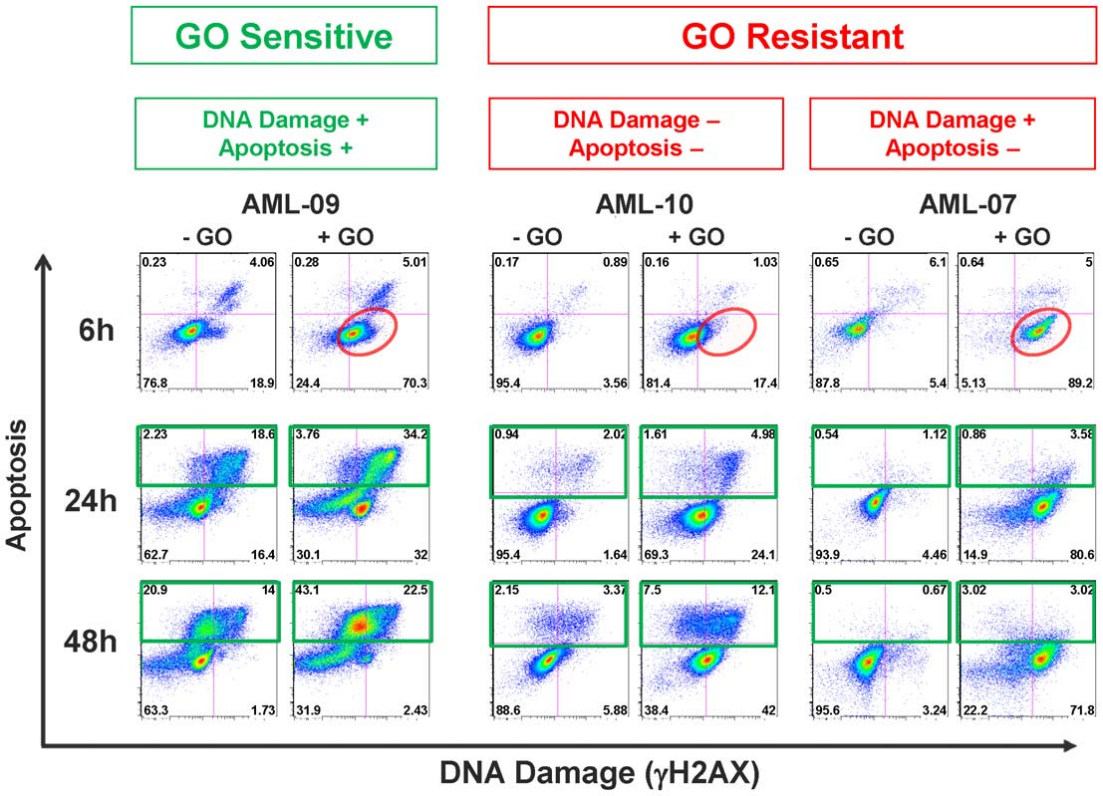
Representative examples of GO sensitive and resistant AML specimens. GO-induced γH2AX (x-axis) and apoptosis (y-axis) at 6 hours (top row), 24 hours (middle row), and 48 hours (bottom row) in specimens AML-09, AML-10, and AML-07, exemplifying the observed response patterns in primary AML. Note: the apoptosis markers shown (y-axis) are cleaved caspase-3 for 6 hour data and cleaved PARP for 24 and 48 hour data; see Figure S2 for a demonstration of concordance between cleaved caspase-3 and cleaved PARP apoptosis markers.

To identify the level at which cellular resistance to GO occurs, we first determined the kinetics of early GO-induced γH2AX in various CD33^+^ human AML cell lines (GDM-1, KG-1, and U937) and peripheral blood mononuclear cells from a healthy donor (Figure S3). These studies indicated robust induction of γH2AX within 6 hours of exposure to GO or GO in combination with a drug efflux inhibitor, cyclosporine A. We therefore chose a 6 hour timepoint to quantify GO-induced DNA damage in individual primary AML specimens. As shown in Table 2, GO induced γH2AX within 6 hours of drug exposure in all primary AML specimens, with the extent of γH2AX induction being highly correlated to CD33 expression levels (r = 0.831 [0.492–0.951], *P* = 0.0008; Figure 2), indicating that CD33 expression is a limiting factor for the intracellular accumulation of the calicheamicin-γ_1_ derivative. In line with this notion, delayed induction of DNA damage was evident by 24 hours in many of the specimens with relatively lower CD33 expression (Table 2, i.e., AML-03, AML-04, AML-05, and AML-10). As the calicheamicin-γ_1_ derivative is a substrate for ATP-binding cassette transporters, we predicted that drug efflux is a second factor that controls GO-induced DNA damage levels. To test this hypothesis, we studied GO-induced γH2AX levels upon co-treatment with the drug efflux inhibitor, PSC833 [32], in the specimens from pediatric patients (limited sample availability precluded testing in specimens from adult patients). We found that PSC833 increased γH2AX induction (at 6 hours: 46.0±10.6% vs. 28.9±11.0%, *P* = 0.0313 [Wilcoxon matched-pairs signed-rank test]), with very marked increases noted in 2/6 specimens (AML-02: increase from 6.8% to 30.9% at 6 hours; and AML-04: increase from 10.9% to 72.9% at 6 hours, respectively) but only minor effects seen in others (AML-05: increase from 23.6% to 31.0% at 6 hours; Figure S4). Together, these findings indicate that both CD33 expression levels and drug efflux play important roles in the extent of GO-induced DNA damage. Nevertheless, while induction of DNA damage appears a prerequisite for GO-induced cytotoxicity, it is not sufficient, as illustrated by 2 specimens in our cohort (AML-07 and AML-08), in which high levels of DNA damage were observed without subsequent apoptosis/cell death (Figure 3), thus suggesting that anti-apoptotic factors downstream of DNA damage are likely important for resistance of these AML cells to GO.

### Association of Induced PI3K/AKT Signaling with in vitro GO Resistance

**Figure 4:**
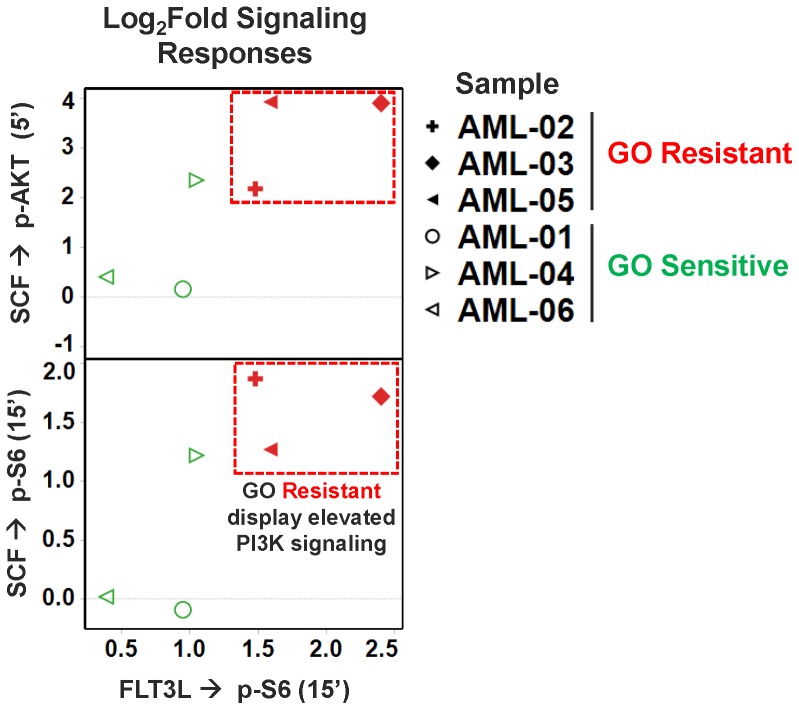
Growth factor/cytokine-induced signaling in primary AML specimens. Plots of signaling log_2_-fold responses: FLT3L induced p-S6 (X-axis) vs. SCF induced p-AKT (top) or SCF induced p-S6 (bottom). Samples are coded by *in vitro* GO response (sensitive: green symbols; resistant: red symbols).

To identify downstream pathways that might affect GO cytotoxicity, we functionally assessed PI3K/AKT/mTOR, JAK/STAT, and Ras/Raf/MEK signaling pathways using SCNP assays; for details regarding modulators and assessed targets, please see Supporting Table S2. Initial experiments in AML cell lines indicated that stem cell factor (SCF)-modulated PI3K pathway activity (as measured by levels of SCF-induced p-AKT and p-S6) was elevated in the GO-resistant GDM-1 but not the GO-sensitive U937 cells (p-AKT: mean log_2_-fold increase of 1.98 in GDM-1 cells vs. −0.11 in U937 cells; for p-S6: mean log_2_-fold increase of 1.00 for GDM-1 cells vs. −0.03 for U937 cells). We then used SCNP assays to assess these signaling pathways in the pediatric AML samples (the adult specimens lacked sufficient material for signaling analyses) using established SCNP methodology for primary human AML [24], [33], [34]. As shown in Figure 4 and Table S3, we found that SCF-modulated PI3K pathway activity was elevated in all 3 GO resistant samples relative to GO sensitive samples (p-AKT: mean log_2_-fold increase of 3.33±0.58 vs. 0.98±0.69, *P* = 0.060; for p-S6: mean log_2_-fold increase of 1.62±0.18 vs. 0.38±0.42, *P* = 0.054). PI3K pathway activity was similarly elevated in GO resistant samples vs. GO sensitive samples when modulated with FLT3 ligand (FLT3L) (p-S6: mean log_2_-fold increase of 1.82±0.29 vs. 0.80±0.21, respectively, *P* = 0.045). Of note, no differences in basal levels p-S6 and p-Akt were observed between GO resistant vs. GO sensitive samples in the absence of SCF or FLT3L (data not shown).

### Effect of AKT Inhibition on GO and Free Calicheamicin-γ_1_-induced Cytotoxicity Against Human AML Cell Lines and Primary AML Cells

**Figure 5:**
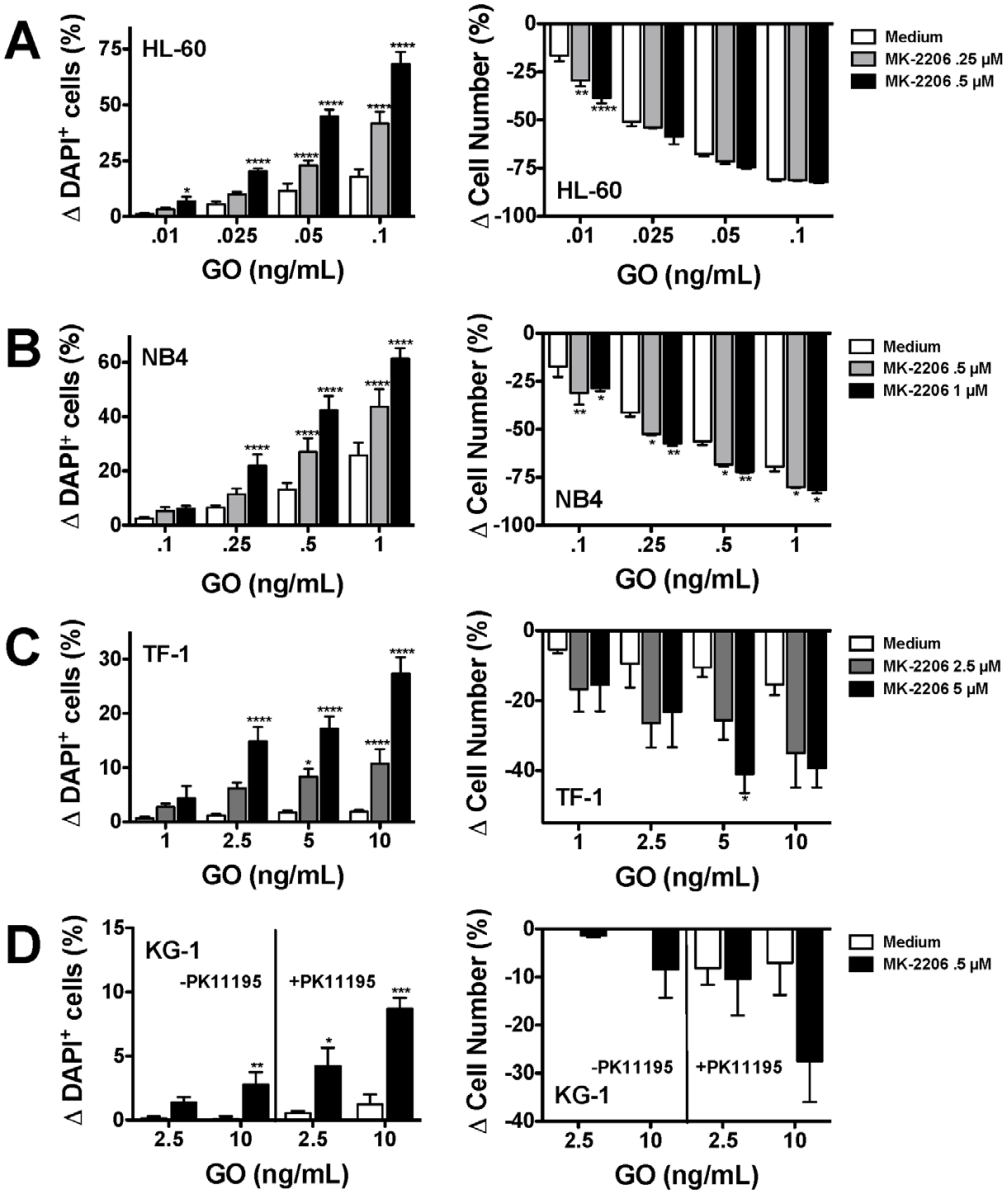
Effect of AKT inhibition on GO-induced cytotoxicity in human AML cell lines *in vitro*. Various doses of the allosteric AKT inhibitor, MK-2206, were incubated with increasing concentrations of GO in (**A**) HL-60, (**B**) NB4, (**C**) TF-1, and (**D**) KG-1 cells. For KG-1 cells, conditions including drug efflux inhibitor PK11195 are also shown. After 3 days, viability (left-side panel) and cell numbers (right-side panel) was determined by flow cytometry. *P<0.05 as compared to medium alone; **P<0.01 as compared to medium alone; ***P<0.001 as compared to medium alone; ****P<0.0001 as compared to medium alone.

**Figure 6:**
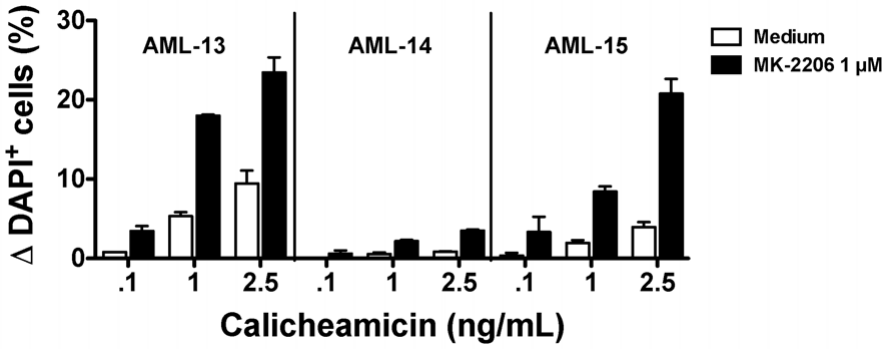
Effect of AKT inhibition on calicheamicin-γ_1_-induced cytotoxicity in primary AML cells *in vitro*. Density gradient-purified mononuclear cells from 3 patients with AML (AML-13, AML-14, and AML-15) were incubated with 2 doses of calicheamicin-γ_1_ in the presence or absence of MK-2206 (1 µM) as indicated. After 3 days, viability was determined by flow cytometry.

Taken together, these data suggest a potential role of PI3K/AKT signaling in the mechanism of resistance to GO or, by extension, calicheamicin-γ_1_-induced cytotoxicity, thus raising the possibility of using inhibitors of this signaling pathway as a novel means to increase the sensitivity of AML cells to calicheamicin-γ_1_-based therapies. To test this hypothesis, we used MK-2206, an investigational small molecule allosteric inhibitor of AKT currently in Phase 2 clinical development for the treatment of various human malignancies including AML. Consistent with previous investigations on solid tumor cancer as well as acute lymphoblastic leukemia cells [35]–[39], treatment with MK-2206 (1 µM for 6 hours) indeed resulted in effective inhibition of S473 AKT phosphorylation while leaving total AKT levels unchanged in human AML cell lines, confirming target inhibition (data not shown). We initially assessed the effect of MK-2206 on GO and calicheamicin-γ_1_-cytotoxicity in various human AML cell lines, which are relatively sensitive (HL-60, NB4) or resistant (TF-1, KG-1) to single agent GO or calicheamicin-γ_1_. As shown in Figure 5, MK-2206 dose-dependently increased *in vitro* GO cytotoxicity in all 4 cell lines, with most marked effects noted in the GO-resistant TF-1 and KG-1 cell lines, suggesting that inhibition of PI3K/AKT survival signaling can sensitize otherwise resistant AML cells to GO-induced cytotoxicity. Of note, in KG-1 cells, which have active drug efflux, MK-2206 significantly sensitized the cells to GO cytotoxicity both in the absence as well as the presence of a drug efflux reversal agent, PK11195, supporting the relevance of PI3K/AKT pathway activation as a limiting factor for GO cytotoxicity even after inhibition of drug efflux. Consistent with the notion that GO-induced cytotoxic effects are primarily due to the calicheamicin-γ_1_ moiety rather than the anti-CD33 antibody, comparable results were obtained when the effect of MK-2206 on cytotoxicity induced by free calicheamicin-γ_1_ was assessed in these cell lines (Figure S5). Finally, to begin investigating the effects of AKT inhibition on GO and calicheamicin-γ_1_-based treatment of primary AML, we treated 3 AML specimens with various doses of calicheamicin-γ_1_ in the presence or absence of a relatively non-toxic dose of MK-2206 (1 µM); limited cell numbers precluded the simultaneous testing of GO in these assays. As shown in Figure 6, the extent of calicheamicin-γ_1_-induced cytotoxicity varied between these 3 specimens; nevertheless, in all 3 cases, MK-2206 enhanced the cytotoxic effects of calicheamicin-γ_1_.

## Discussion

Clinical studies conducted over the last decade have unequivocally demonstrated that GO provides clinical benefit in a subset of patients with AML while being largely ineffective in others [5]. Therefore, the ability to prospectively identify patients whose leukemias are resistant to GO, and unraveling the mechanisms underlying this resistance, would provide an important tool to optimize the clinical use of this antibody-drug conjugate. By simultaneously measuring multiple elements associated with cellular resistance to GO, including target antigen expression, drug-efflux mechanisms, extent of DNA damage, and cellular signaling networks, we show that SCNP assays offer a hypothesis-generating approach to discover novel biological features associated with GO sensitivity or resistance of human AML cells *in vitro*.

Using SCNP assays, the data from the current study support three major conclusions. First, DNA damage is required but not sufficient for GO-induced cytotoxicity. This finding is consistent with genotoxic responses previously observed in AML samples treated with other agents such as etoposide or cytarabine/daunorubicin [24], [40], and provides direct evidence for the significance of anti-apoptotic factors downstream of DNA damage as resistance factors to GO. Future studies on larger numbers of primary AML specimens will be required to test whether such anti-apoptotic mechanisms are indeed more relevant in adult AML, as perhaps suggested by our small dataset. Importantly, both CD33 expression levels and drug efflux affect the extent of GO-induced DNA damage, confirming previous studies demonstrating that these factors are pivotal determinants affecting the final intracellular accumulation of the calicheamicin-γ_1_ derivative [13]. The dependence of *in vitro* GO cytotoxicity on sufficient DNA damage, as determined by induced γH2AX levels, suggests that lack of γH2AX induction by GO could serve as an early biomarker of resistance to GO that could be available within hours after exposure to GO.

Second, our data are the first to indicate that anti-apoptotic pathways downstream of DNA damage contribute to *in vitro* cellular resistance to GO in primary AML samples. This finding supports a model in which the toxicity of the calicheamicin-γ_1_ moiety is critically modulated by the ability of the cell to repair DNA damage as well as the activity of downstream pro- and anti-apoptotic pathways, and may offer a partial biological explanation for the observation that calicheamicin-γ_1_ *in vitro* sensitivity varies over 100 000-fold among individual primary AML cell samples [41].

Third, and most importantly, our studies identify activated PI3K/AKT signaling, measured after growth factor treatment, as a novel mechanism of resistance to GO and, by extension, calicheamicin-γ_1_. AKT is downstream of PI3K and the primary effector molecule of the PI3K signaling cascade [8]. After activation via phosphorylation and translocation to the nucleus, AKT directly or indirectly affects the activity of several transcription factors. Accumulating evidence demonstrates that the PI3K/AKT signaling axis is crucial to many aspects of cell growth, survival, and apoptosis [8]. In AML, recent studies have demonstrated that PI3K/AKT signaling is frequently activated [42], [43], and activated AKT is associated with poor outcome [44]. Of note, in a recent study, in which SCNP assays were used to develop classifiers for the prediction of clinical response to standard induction therapy in AML, elevated PI3K pathway signaling was consistently associated with therapeutic failure [16]. Based on these data, AKT has been identified as a rational drug target in AML. Our *in vitro* data with MK-2206 indicate that AKT signaling modulates GO/calicheamicin-γ_1_ cytotoxicity and is associated with cellular resistance to these drugs. In turn, inhibition of AKT activation can greatly increase GO/calicheamicin-γ_1_ sensitivity, suggesting a role of AKT inhibitors such as MK-2206 as chemosensitizers for GO or, more generally, calicheamicin-γ_1_-based treatment strategies. Further studies will be required to fully understand the mechanistic details by which activation of this pathway interferes with GO/calicheamicin-γ_1_-induced cytotoxicity.

Together, by identifying an association between PI3K/AKT signaling activation and *in vitro* GO and calicheamicin-γ_1_ resistance, our data illustrate the capacity of SCNP assays to functionally characterize the integrity of biological pathways and signaling networks relevant to cellular responses to genotoxins such as calicheamicin-γ_1_-based antibody-drug conjugates *in vitro* in primary samples from patients with AML. Although future studies with larger numbers of patient specimens will be required to validate our studies and quantify the contribution of AKT-mediated resistance to GO and calicheamicin-γ_1_ in detail, our data highlight the potential of SCNP assays to differentiate AML samples based on underlying biology which might be relevant to therapeutic interventions. Our data form the basis for future studies in which such assays are applied to AML samples from patients with documented clinical outcomes to validate findings from *in vitro* predictions. If validated, SCNP assays could serve to prospectively identify subsets of patients that are suitable for GO-based therapy – a critical advancement toward optimized drug use – and may help in the discovery of additional therapeutic targets,. By monitoring multiple cellular characteristics (e.g. signaling in several survival pathways), SCNP assays may provide an ideal tool to prospectively inform about multiple GO-relevant resistance mechanisms simultaneously, and could help in the selection of the most appropriate chemosensitizing agents and drug combination strategies in patients under consideration for GO-containing therapy, thus further contributing to the individualization of AML treatment approaches.

## Supporting Information

Figure S1 **Quantification of CD33 Qdot605 MFI in primary AML specimens.** To compute the equivalent CD33 MFI value in the Qdot605 scale for sample AML-11 (which lacked this data point), a linear regression model was constructed comparing CD33 Qdot605 MFI with CD33 PE MFI and used to compute the equivalent Qdot605 MFI value for AML-11 based on its CD33 PE MFI value. AML samples and peripheral blood and bone marrow mononuclear cells from normal donors (from our internal database) with available data on both fluorophores (left table), was used to calculate a linear regression model between the CD33 Qdot605 and PE MFI scales (middle), allowing for computation of the equivalent CD33 Qdot605 MFI in specimen AML-11 using the equation: Qdot605-CD33 MFI  = 0.6927×(CD33-PE MFI) +176.05 (r = 0.94) (right).
(TIFF)

Figure S2 **Effect of staurosporine on GO-resistant pediatric AML samples.** Primary AML specimens were treated with staurosporine for 6 hours prior to flow cytometric assessment of apoptosis using cleaved caspase-3 (X-axis) and cleaved PARP (Y-axis). All GO-resistant samples except AML-10 showed robust apoptotic responses, as demonstrated by presence of >60–90% double positive cells upon treatment with staurosporine. Note: the apoptosis markers, cleaved caspase-3 and cleaved PARP, showed concordant results with an average of >90% of cells being either double positive or double negative for these markers.
(TIFF)

Figure S3 **Kinetics of GO-induced DNA damage response** ***in vitro*** **.** (A) Kinetics of GO-induced DNA damage measured by γH2AX levels. Treatment conditions are indicated by shape: GO alone (squares), drug efflux inhibitor (cyclosporine A, CsA) alone (circles), and GO in combination with cyclosporine A (crosses),.AML cell lines GDM-1 and KG-1 (left panels) were resistant to GO treatment alone with minimal induction of DNA damage at any time-points tested; however, U937 cells and primary myeloid PBMC (right panels) were sensitive to GO treatment alone with highest γH2AX levels observed at 6–8 hours. Co-treatment of GO with cyclosporine A sensitized MDR+ KG-1 and GDM-1 cells to GO-induced DNA damage.
(TIFF)

Figure S4 **Inhibition of drug efflux activity by PSC833 increases GO sensitivity in some AML samples.** Drug transporter activity was assessed by co-treatment of GO and the pump efflux inhibitor, PSC833. DNA damage (γH2AX, X-axis) and apoptosis (cleaved PARP, Y-Axis) responses are shown for AML-02 (left) and AML-05 (right) in the presence of GO alone, PSC833 alone, or GO in combination with PSC833. While PSC833 co-treatment substantially increased GO induced apoptosis in AML-02, PSC833 co-treatment did not sensitize AML-05 to GO induced apoptosis. Circles highlight γH2AX+ cells (red) and apoptotic cPARP+ cells (green).
(TIFF)

Figure S5 **Effect of AKT inhibition on calicheamicin-γ_1_-induced cytotoxicity in human AML cell lines** ***in vitro*** **.** Various doses of MK-2206 were incubated with increasing concentrations of calicheamicin-γ_1_ in **(A)** HL-60, **(B)** NB4, **(C)** TF-1, and **(D)** KG-1 cells. After 3 days, viability (left-side panel) and cell numbers (right-side panel) was determined by flow cytometry. *P<0.05 as compared to medium alone; **P<0.01 as compared to medium alone; ***P<0.001 as compared to medium alone; ****P<0.0001 as compared to medium alone.
(TIFF)

Table S1 **List of reagents.**
(TIFF)

Table S2 **Conditions and staining cocktails used in SCNP signaling assays.**
(TIFF)

Table S3 **Log_2_Fold signaling responses of AML samples.**
(TIFF)
