# Supplementary figures and images for: AKT Signaling as a Novel Factor Associated with In Vitro Resistance of Human AML to Gemtuzumab Ozogamicin
Source: PLoS One. 2013 Jan 8;8(1):e53518. doi: 10.1371/journal.pone.0053518 (PMC3539972; doi:10.1371/journal.pone.0053518)

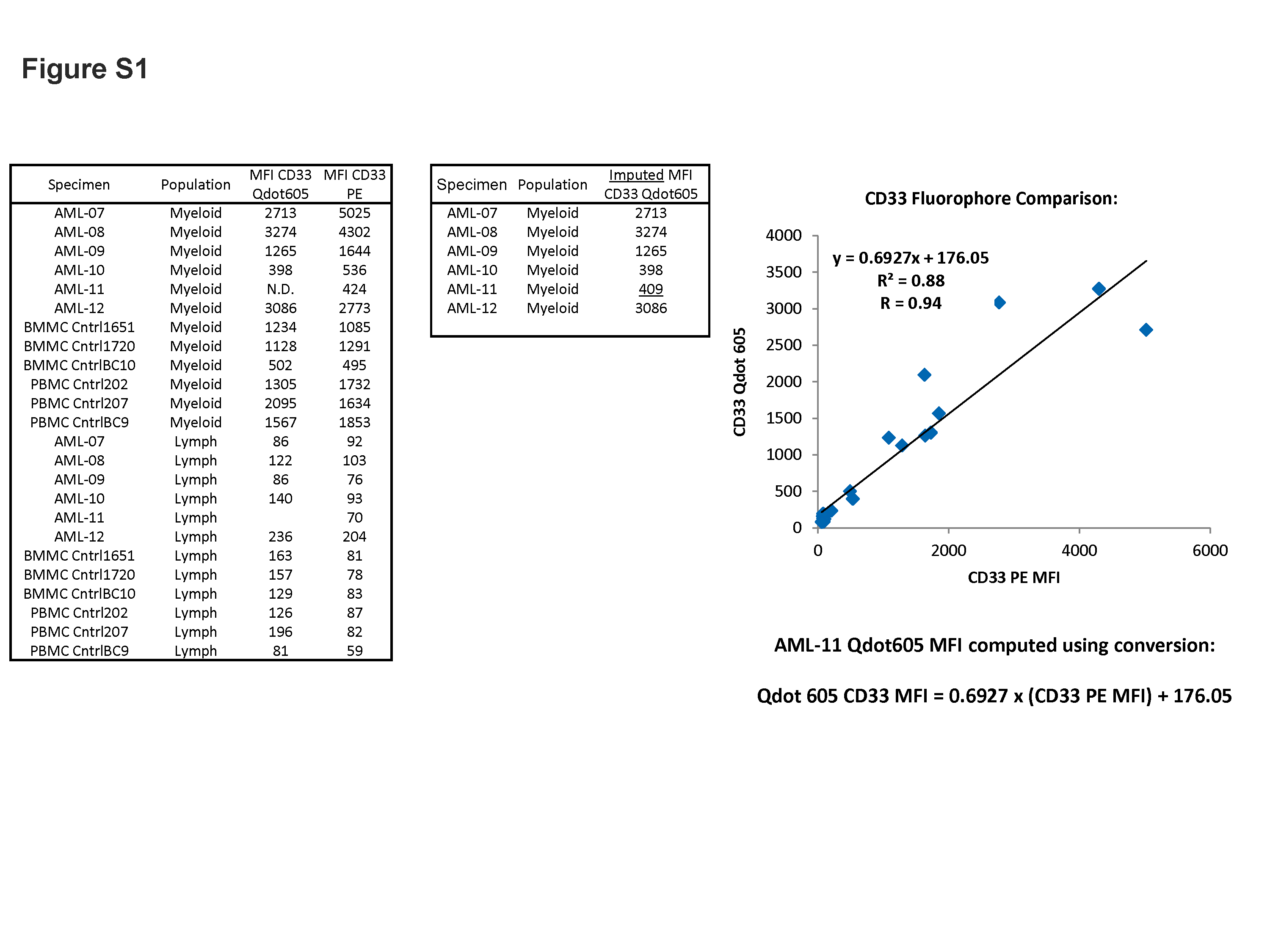

Supplement: Figure S1 — Quantification of CD33 Qdot605 MFI in primary AML specimens. To compute the equivalent CD33 MFI value in the Qdot605 scale for sample AML-11 (which lacked this data point), a linear regression model was constructed comparing CD33 Qdot605 MFI with CD33 PE MFI and used to compute the equivalent Qdot605 MFI value for AML-11 based on its CD33 PE MFI value. AML samples and peripheral blood and bone marrow mononuclear cells from normal donors (from our internal database) with available data on both fluorophores (left table), was used to calculate a linear regression model between the CD33 Qdot605 and PE MFI scales (middle), allowing for computation of the equivalent CD33 Qdot605 MFI in specimen AML-11 using the equation: Qdot605-CD33 MFI = 0.6927×(CD33-PE MFI) +176.05 (r = 0.94) (right). (TIFF) [file pone.0053518.s001.tiff]

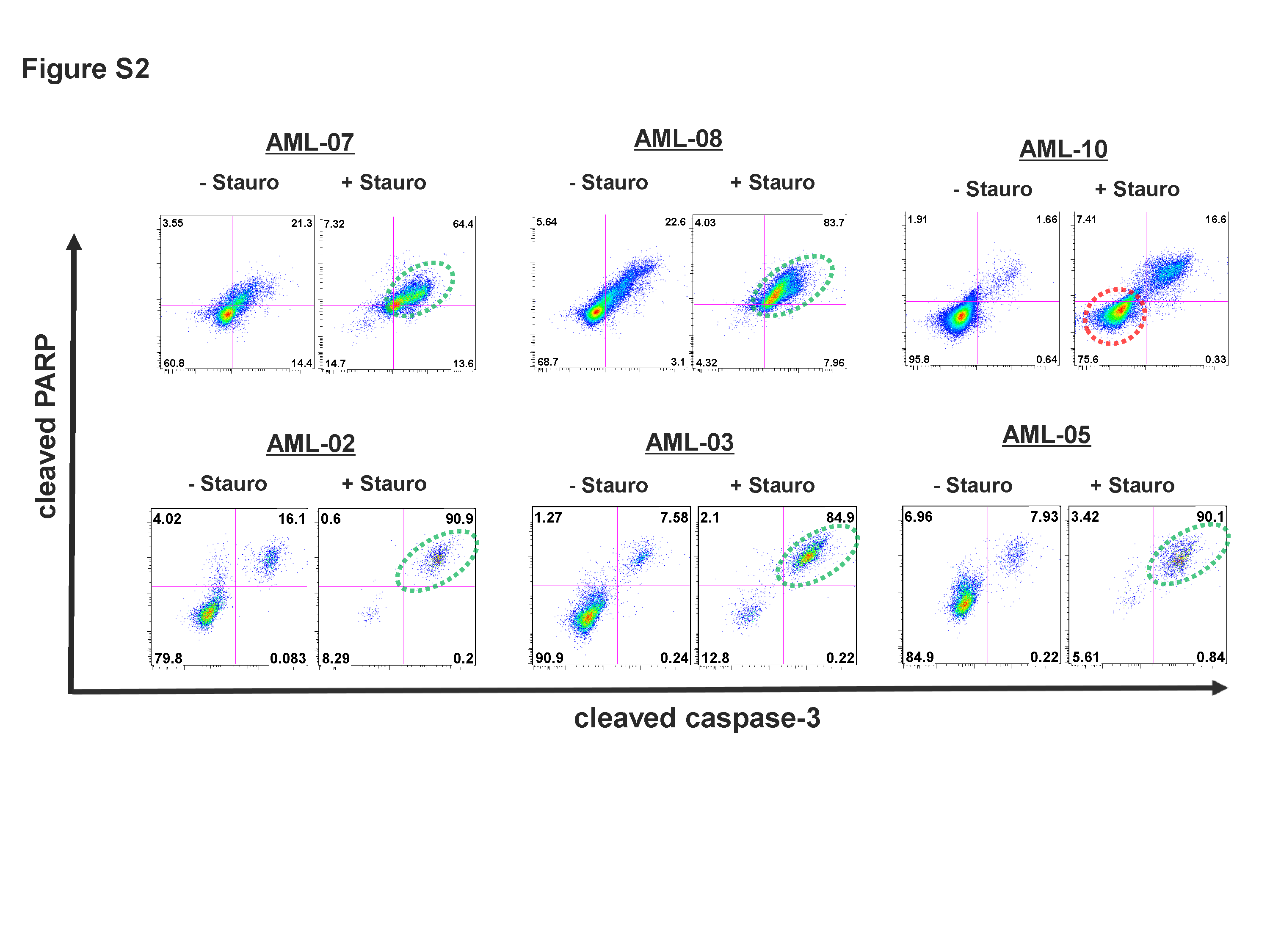

Supplement: Figure S2 — Effect of staurosporine on GO-resistant pediatric AML samples. Primary AML specimens were treated with staurosporine for 6 hours prior to flow cytometric assessment of apoptosis using cleaved caspase-3 (X-axis) and cleaved PARP (Y-axis). All GO-resistant samples except AML-10 showed robust apoptotic responses, as demonstrated by presence of >60–90% double positive cells upon treatment with staurosporine. Note: the apoptosis markers, cleaved caspase-3 and cleaved PARP, showed concordant results with an average of >90% of cells being either double positive or double negative for these markers. (TIFF) [file pone.0053518.s002.tiff]

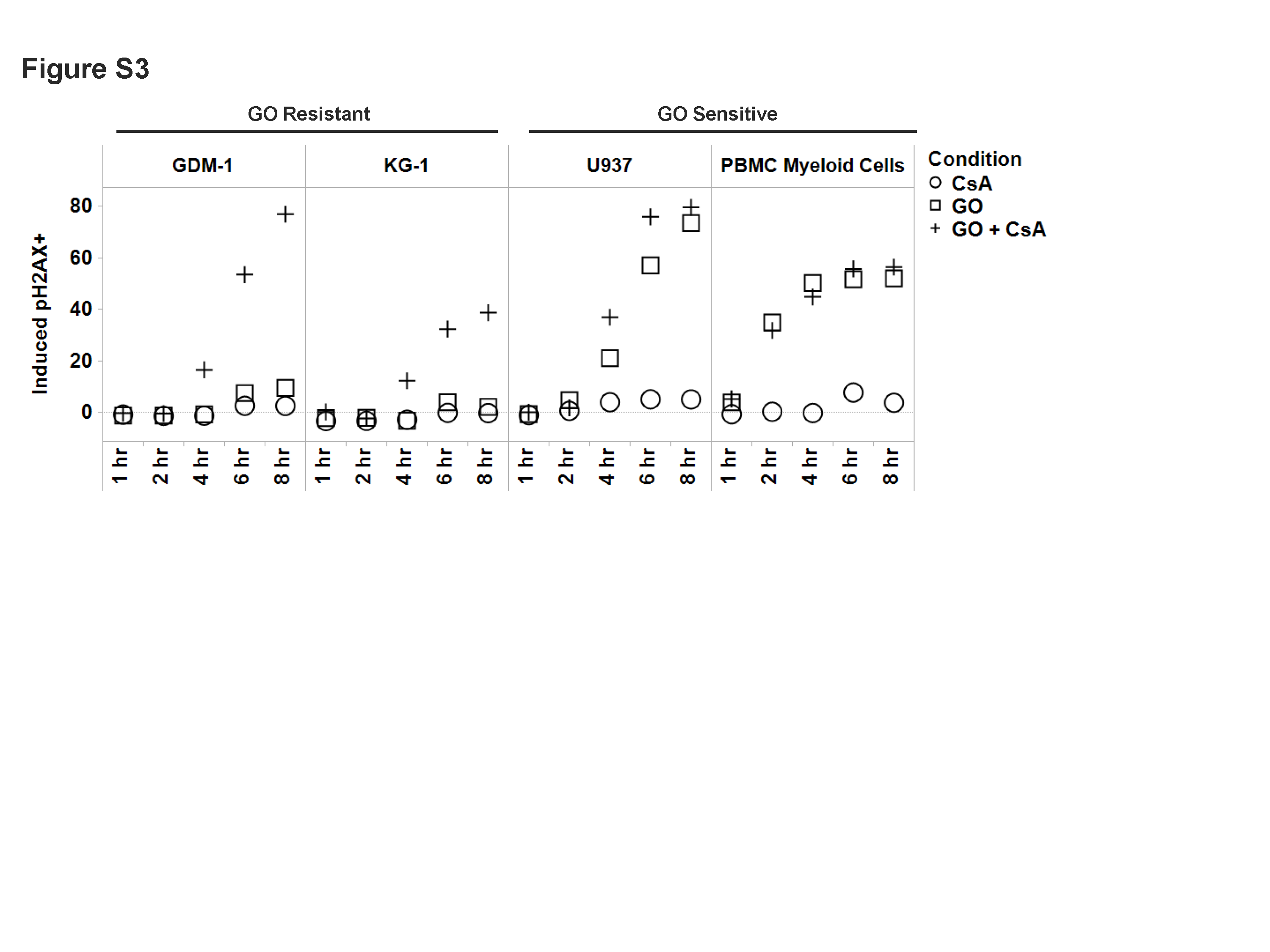

Supplement: Figure S3 — Kinetics of GO-induced DNA damage response in vitro . (A) Kinetics of GO-induced DNA damage measured by γH2AX levels. Treatment conditions are indicated by shape: GO alone (squares), drug efflux inhibitor (cyclosporine A, CsA) alone (circles), and GO in combination with cyclosporine A (crosses),.AML cell lines GDM-1 and KG-1 (left panels) were resistant to GO treatment alone with minimal induction of DNA damage at any time-points tested; however, U937 cells and primary myeloid PBMC (right panels) were sensitive to GO treatment alone with highest γH2AX levels observed at 6–8 hours. Co-treatment of GO with cyclosporine A sensitized MDR+ KG-1 and GDM-1 cells to GO-induced DNA damage. (TIFF) [file pone.0053518.s003.tiff]

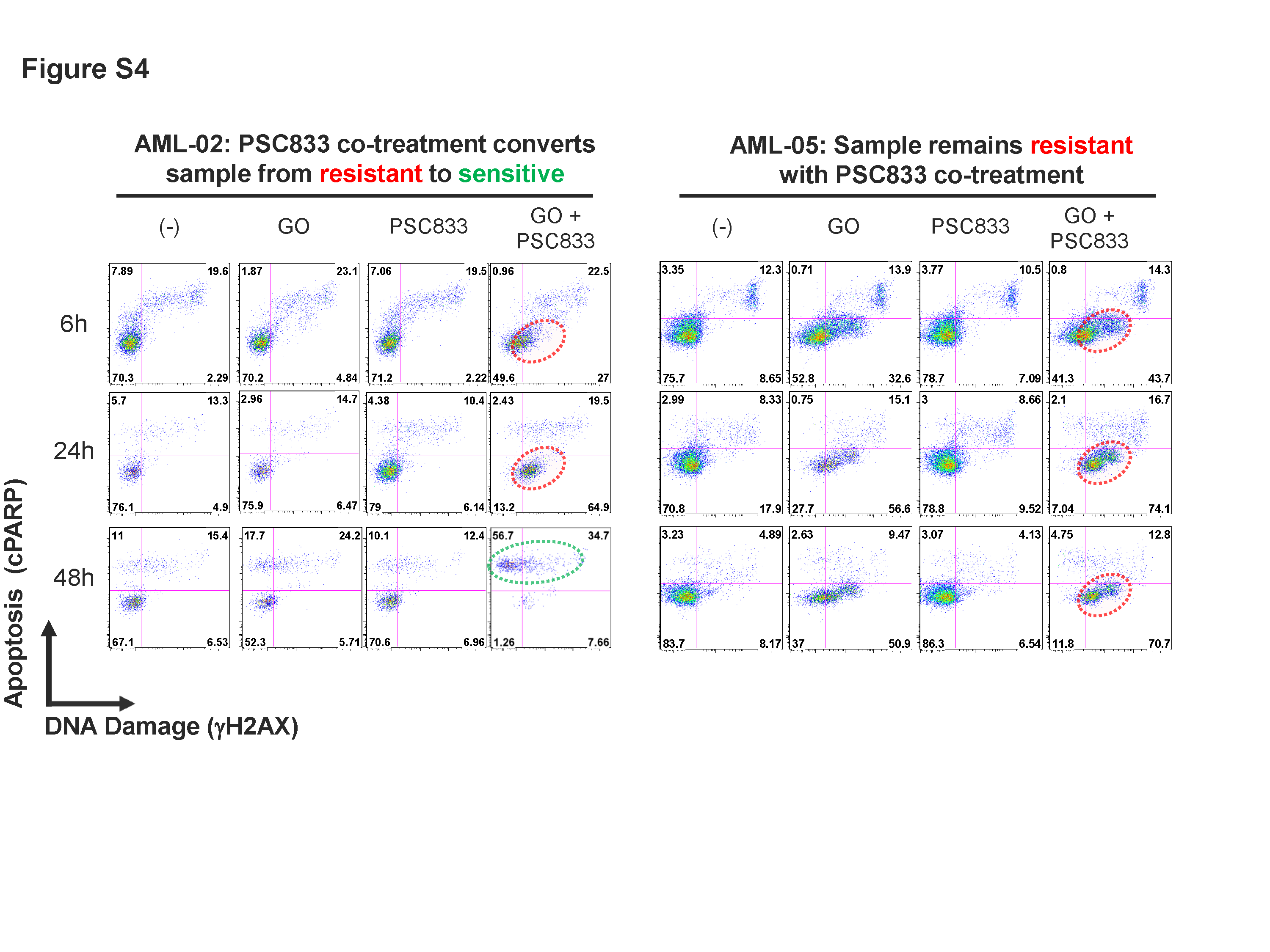

Supplement: Figure S4 — Inhibition of drug efflux activity by PSC833 increases GO sensitivity in some AML samples. Drug transporter activity was assessed by co-treatment of GO and the pump efflux inhibitor, PSC833. DNA damage (γH2AX, X-axis) and apoptosis (cleaved PARP, Y-Axis) responses are shown for AML-02 (left) and AML-05 (right) in the presence of GO alone, PSC833 alone, or GO in combination with PSC833. While PSC833 co-treatment substantially increased GO induced apoptosis in AML-02, PSC833 co-treatment did not sensitize AML-05 to GO induced apoptosis. Circles highlight γH2AX+ cells (red) and apoptotic cPARP+ cells (green). (TIFF) [file pone.0053518.s004.tiff]

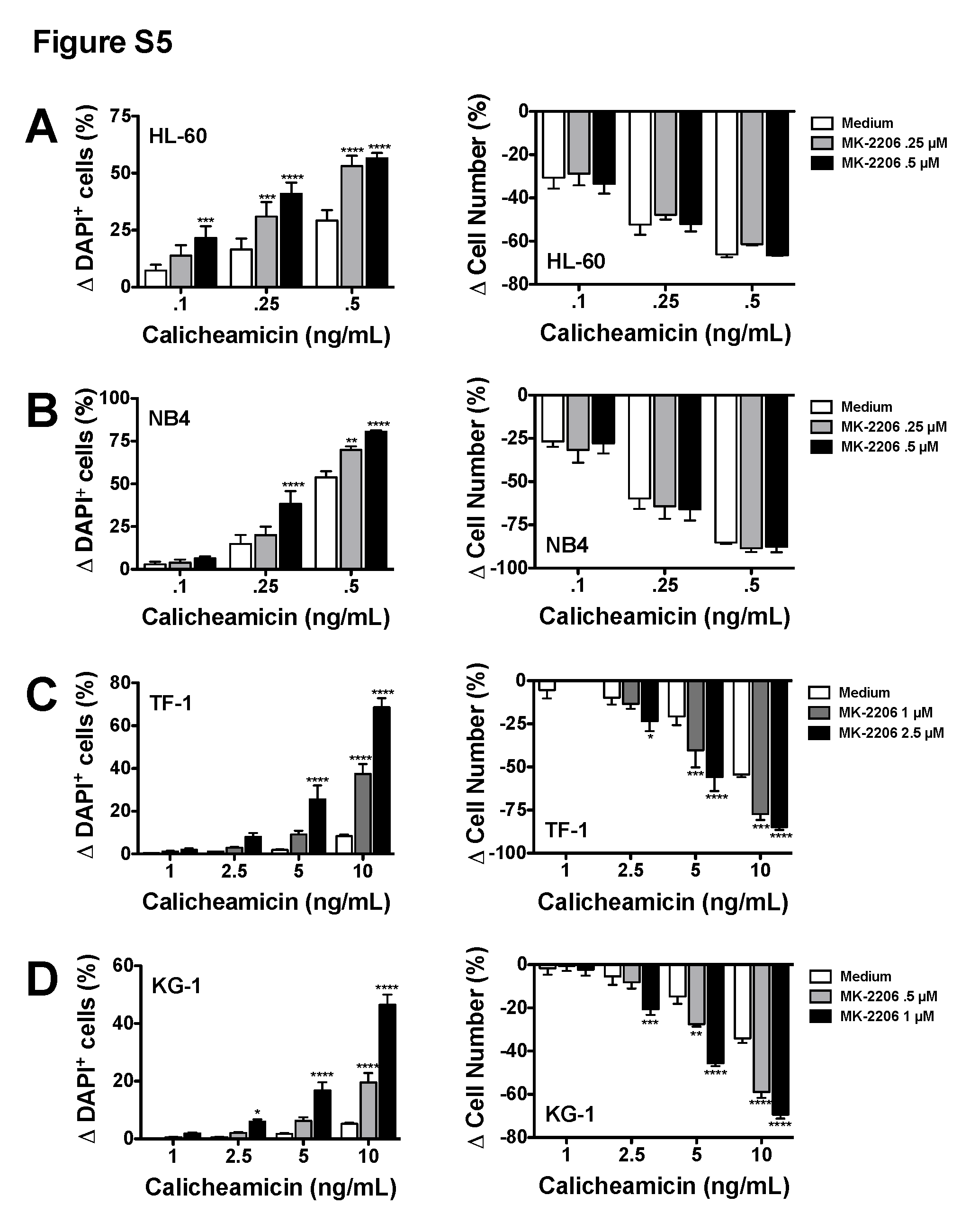

Supplement: Figure S5 — Effect of AKT inhibition on calicheamicin-γ1-induced cytotoxicity in human AML cell lines in vitro . Various doses of MK-2206 were incubated with increasing concentrations of calicheamicin-γ1 in (A) HL-60, (B) NB4, (C) TF-1, and (D) KG-1 cells. After 3 days, viability (left-side panel) and cell numbers (right-side panel) was determined by flow cytometry. *P<0.05 as compared to medium alone; **P<0.01 as compared to medium alone; ***P<0.001 as compared to medium alone; ****P<0.0001 as compared to medium alone. (TIFF) [file pone.0053518.s005.tiff]

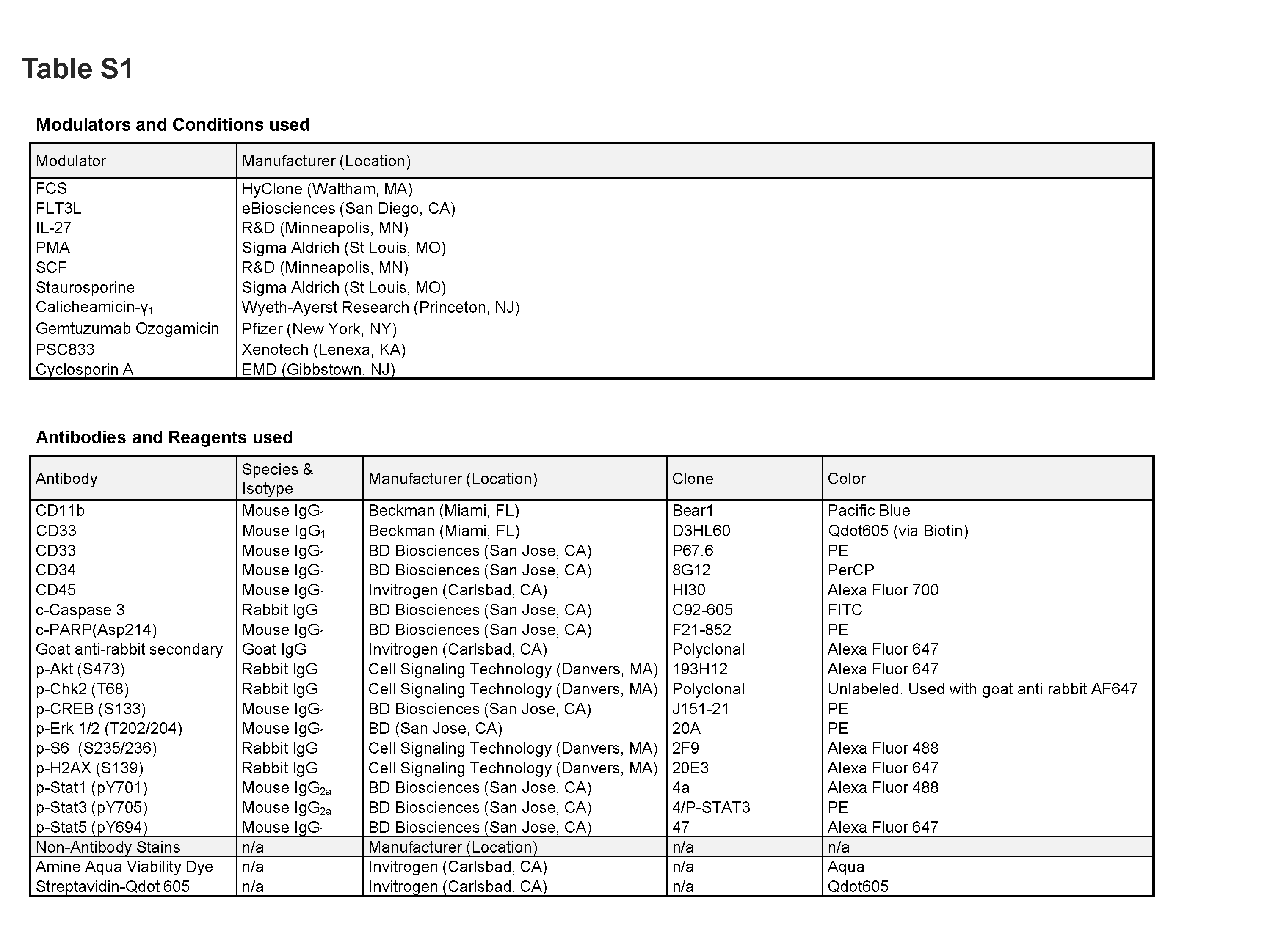

Supplement: Table S1 — List of reagents. (TIFF) [file pone.0053518.s006.tiff]

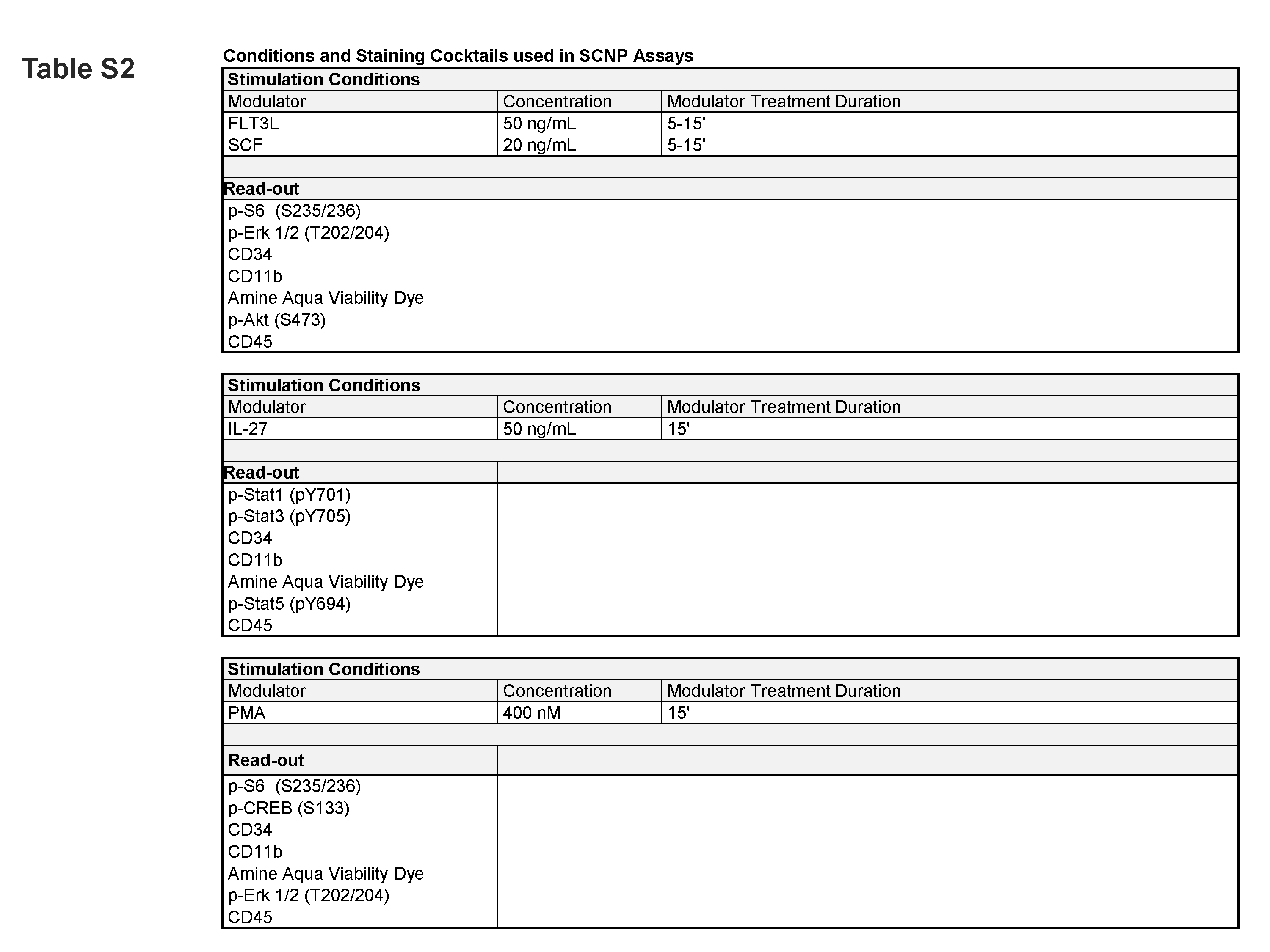

Supplement: Table S2 — Conditions and staining cocktails used in SCNP signaling assays. (TIFF) [file pone.0053518.s007.tiff]

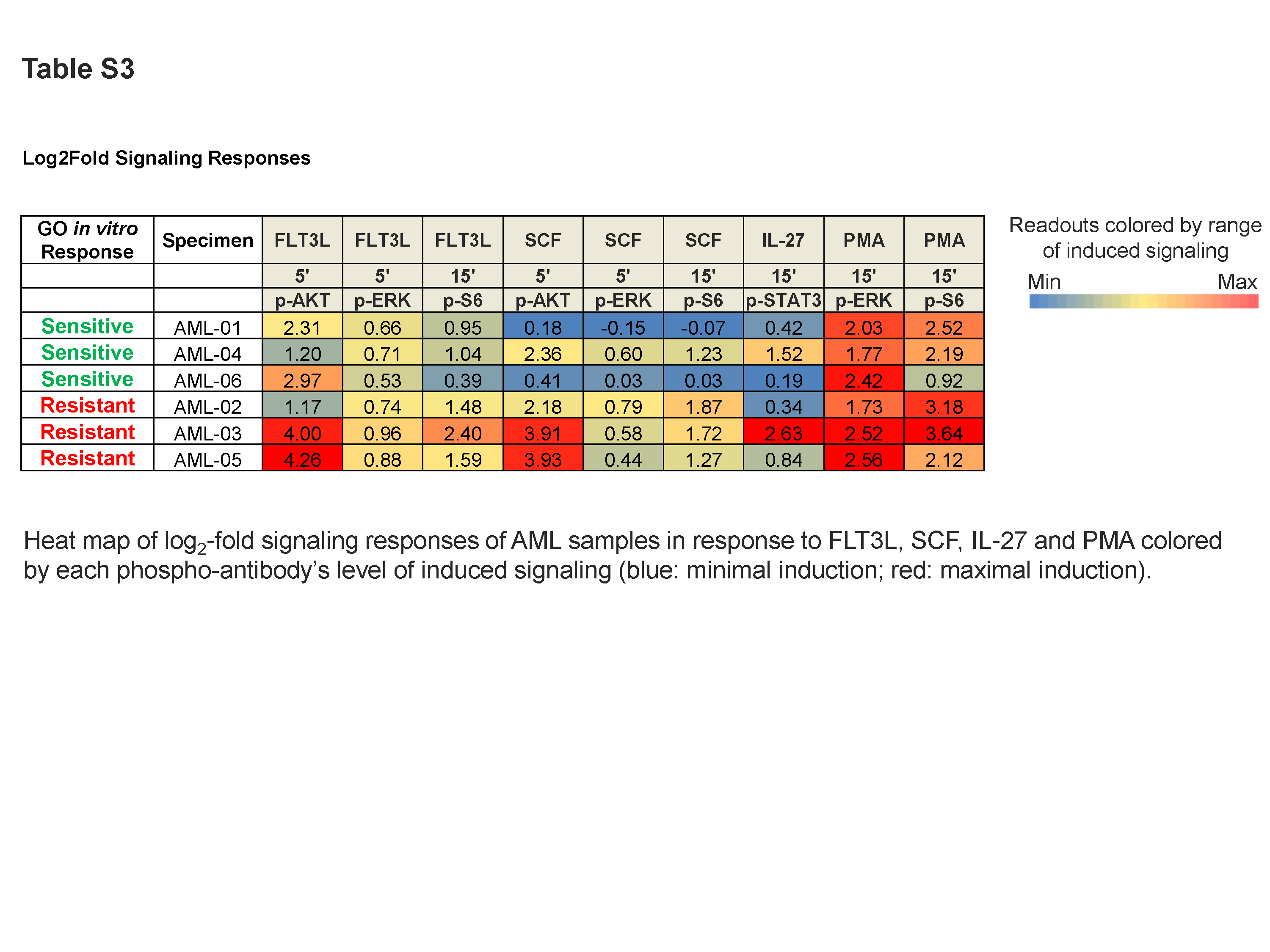

Supplement: Table S3 — Log2Fold signaling responses of AML samples. (TIFF) [file pone.0053518.s008.tiff]
